# Measuring the Dispositional Tendency to Spread Oneself Too Thin

**DOI:** 10.3389/fpsyg.2018.02549

**Published:** 2018-12-13

**Authors:** Louis Boemerman, Lauren Kuykendall

**Affiliations:** ^1^Department of Psychology, Pennsylvania State University, University Park, PA, United States; ^2^Department of Psychology, George Mason University, Fairfax, VA, United States

**Keywords:** work-nonwork conflict, work-family conflict, spreading oneself too thin, self-enhancement, scale validation

## Abstract

This paper describes the development and validation of a scale to measure the dispositional tendency to spread oneself too thin (SOTT) – a tendency that is likely an important antecedent of work-nonwork conflicts. In two studies, we develop and validate a scale for measuring this tendency and examine how it relates to other dispositional causes of role conflicts. In Study 1 we tested our initial item pool using a heterogeneous sample of full-time workers (*N* = 193) and used exploratory factor analysis to reduce our item pool to a set of five items. In Study 2, using another heterogeneous sample of full-time workers (*N* = 212), we conducted confirmatory factor analyses to demonstrate that the items fit a unidimensional construct. We also demonstrated that the scale has moderately high test-retest reliability, is distinct from other potentially related constructs, and predicts work-nonwork conflicts above and beyond previously studied dispositional antecedents. We discuss the importance of studying the dispositional tendency to SOTT as a potentially malleable antecedent of work-nonwork conflict and note other employee outcomes that it may also impact.

## Introduction

While it is not uncommon to hear a person attribute his or her work-nonwork conflict to “spreading myself too thin” or “biting off more than I can chew,” this tendency has not yet been discussed in the work-nonwork literature. Drawing on the lexical hypothesis (Allport and Odbert, 1936), which posits that important psychological characteristics are encoded into language, we suggest that the common use of phrases such as “spreading myself too thin” and “biting off more than I can chew” points to an important psychological characteristic. The goal of this paper is to better understand this characteristic and incorporate it into the work-nonwork literature. Specifically, we develop and validate a self-report scale to measure the dispositional tendency to spread oneself too thin (SOTT) and examine its effects on work-nonwork conflict. In doing so, we respond to calls for further research into novel dispositional predictors of work-nonwork conflicts (Allen, 2012).

We define the dispositional tendency to SOTT as *the tendency to commit oneself to more projects and/or activities than one can effectively manage*. This tendency is likely a result of self-enhancement biases (e.g., unrealistic optimism and overconfidence; Greenwald, 1980; Weinstein, 1982), which can cause individuals to adopt and pursue unrealistic goals because of the desire to hold positive self-views. While these biases are thought to be pervasive, some individuals are likely more prone to such biases than others (Oreg and Bayazit, 2009). Given that individuals with this tendency are likely to commit themselves to more than they can effectively accomplish across numerous life roles (i.e., work roles, family roles, personal roles), this tendency should lead to role conflicts, as individuals struggle to accomplish more than is realistically possible in and across roles.

We conducted two studies to develop and validate a measure for the dispositional tendency to SOTT. In Study 1, we designed and tested the initial group of items created for measuring this tendency. In Study 2, we completed confirmatory analyses on the items and found evidence for their reliability and validity.

## Study 1

### Methods

#### Item Generation

We followed the procedures outlined by Hinkin (1998) to create a set of 15 items (shown in Table 1) that captured the dispositional tendency to SOTT. Items were then reviewed by two students and one faculty member for clarity and representativeness of the construct.

**Table 1:** Means, standard deviations, and factor loadings from a pattern matrix of the dispositional tendency to spread oneself too thin items.

Item	Mean	SD	5-item factor loading	15-item factor loading
**Final items**				
I sometimes feel like I “bite off more than I can chew”	3.53	1.65	0.89	0.89
I sometimes feel like I “spread myself too thin”	3.68	1.67	0.86	0.84
I have a tendency to be involved in too many different projects	3.72	1.61	0.88	0.87
I have a tendency to take on more activities than I have time for	3.74	1.64	0.91	0.90
I have a tendency to take on more than I can handle	3.62	1.63	0.91	0.89
**Dropped items**				
Sometimes I take on commitments I cannot fulfill	3.41	1.61		0.79
I rarely have enough time to accomplish everything I set out to	3.69	1.61		0.77
Others have told me I have a tendency to “bite off more than I can chew”	3.46	1.65		0.85
I have been told I have a habit of “spreading myself too thin”	3.48	1.67		0.84
People have told me I sometimes take on too many projects at once	3.58	1.65		0.87
I tend to start more projects than I finish	3.41	1.69		0.78
I often feel overcommitted	3.60	1.68		0.82
Sometimes I take on more than I can handle	3.68	1.68		0.87
No matter how much I already have going on, it is hard for me not to get involved in new projects	3.76	1.57		0.73
I have been known to start new projects even when my schedule is already full	3.78	1.61		0.85


*Factor loadings are derived from a pattern matrix. Eigenvalue = 10.53. Percentage Variance Explained = 70.232.*

#### Participants

We tested our initial item pool using a heterogeneous sample of full-time workers recruited via Amazon Mechanical Turk (MTurk). The sample included workers from a variety of industries, most commonly healthcare and social assistance (10.9%) and retail (11.4%); 47% of participants were female; 70.9% were White; and 44.6% reported having at least a bachelor’s degree. The median age was 31 years old (*SD* = 7.92).

#### Procedures

We asked participants to indicate the degree to which the 15 statements in our item pool accurately describe them (1 = *strongly disagree* to 7 = *strongly agree*).

### Results

#### Analyses and Item Reduction

An exploratory factor analysis was conducted with principal components analysis and a direct oblimin rotation. Eigenvalues and scree plots suggested that a one-dimensional structure best fit the data. All items loaded onto this single factor, shown in Table 2. We sought to shorten the length of our measure to an ideal length of four to six items using a combination of internal item qualities (e.g., factor loadings) and judgmental item qualities (e.g., clarity, face validity, and non-redundancy) (Stanton et al., 2002). All items had high factor loadings (>0.70); therefore, no items were eliminated based on low loadings. To reduce items, coders identified sets of items that they agreed were semantically similar and retained the item that had the highest factor loading. In cases where two items had an equally high loading, the coders retained the items that they deemed to have a more commonplace wording. Table 1 shows the initial 15 items and final five items (α = 0.95), along with means, standard deviations, and factor loadings. We acknowledge that, by including colloquial terms (“bite off more than I can chew”), our survey may not be valid when used by non-native English speakers. While we note this as an important limitation, we felt that including these items was the most face valid way to accurately assess the phenomenon.

**Table 2:** Incremental effects of the dispositional tendency to spread oneself too thin on role conflict.

	Work-personal conflict	Personal-work conflict	Work-family conflict	Family work conflict	Family personal conflict	Personal-family conflict
	
	**B**	**B**	**B**	***B***	**B**	**B**
**Step 1**						
Negative affect	0.36^∗∗^	0.47^∗^	0.50^∗^	0.45^∗^	0.27^∗^	0.46^∗^
Neuroticism	0.23^∗^	0.05	0.15^∗^	0.02	0.10	-0.06
Δ*R*^2^	0.26^∗∗^	0.22^∗^	0.27^∗^	0.17^∗^	0.10^∗^	0.21^∗^
**Step 2**						
Dispositional tendency to spread oneself too thin	0.17^∗∗^	0.05	0.18^∗^	0.08^∗^	0.17^∗^	0.04
Δ*R*^2^	0.06^∗∗^	0.01	0.06^∗^	0.02^∗^	0.05^∗^	0.00
Total *R*^2^	0.32	0.23	0.33	0.19	0.15	0.21
Adjusted *R*^2^	0.31	0.22	0.32	0.18	0.14	0.20


*N = 212. ^∗^p < 0.05, ^∗∗^p < 0.01.*

## Study 2

### Methods

The goals for Study 2 were as follows: (1) to conduct a confirmatory analysis on the items and assess the psychometric qualities of the scale and (2) to determine the discriminant, incremental, and criterion-related validity of our scale.

### Examining Validity

#### Discriminant Validity

It is important to distinguish the tendency to SOTT from several similar variables. First, SOTT must be distinguished from role overload which refers to employees feeling that “there are too many responsibilities or activities expected of them in light of the time available, their abilities, and other constraints” (Bolino and Turnley, 2005, p. 741). While both role overload and SOTT involve feeling like one has more to do than can be effectively managed, role overload is typically understood as a function of situational demands or other’s expectations, whereas the dispositional tendency to SOTT involves an individual choosing to take on more than he or she can effectively manage. Additionally, a trait that SOTT needs to be distinguished from is achievement striving, a sub-dimension of the Five Factor personality trait conscientiousness (Costa et al., 1991). Achievement striving is defined as “a striving for excellence” (Costa et al., 1991, p. 889). Achievement striving is distinct from the dispositional tendency to SOTT because, while both involve the tendency to pursue goals, achievement does not necessarily involve a disposition to pursue more goals than one can handle. The dispositional tendency to SOTT must also be differentiated from the personality trait agreeableness – the tendency to be kind, cooperative, and considerate (Thompson, 2008). It is easy to imagine a highly agreeable individual taking on more than he or she can handle out of a desire to please others. However, while the desire to please others may be one motivating factor explaining the dispositional tendency to SOTT, there are many other factors that may contribute. We posit that agreeableness may be an antecedent of the dispositional tendency to SOTT, but is not a redundant construct.

#### Criterion-Related Validity and Incremental Validity

To assess criterion-related validity, we assessed correlations between our scale and work-nonwork conflicts (i.e., conflicts among work, family, and personal roles). To assess incremental validity, we assessed the extent to which our scale predicts work-nonwork conflicts beyond the strongest known negative dispositional predictors (i.e., neuroticism and negative affectivity; Allen et al., 2012).

#### Participants

We used the same recruitment approach as in Study 1, recruiting full time working participants from Amazon Mechanical Turk (MTurk). Two-hundred and eighteen participants (none of whom participated in Study 1) were recruited. Six participants were disqualified because they failed attention check items. All participants were full-time workers. The sample included workers from a variety of industries, most commonly financial and insurance (10.7%) and real estate (10.7%) – 47% of participants were female; 74.8% were White, and 37.4% reported having at least a bachelor’s degree. Thirty-three years old was the median age (*SD* = 10.14).

#### Procedures

We conducted this study in two waves, following up with participants 3 months after initial data collection in order to establish test-retest reliability. The dispositional tendency to SOTT measure was administered before any work related measures, in order to avoid biasing participants into viewing it as a work-specific construct. Eighty-three participants completed the second survey.

### Measures

#### Negative Affect

Negative affect was measured using the 10-item subset of the Positive and Negative Affect Schedule (Watson and Clark, 1999).

#### Role Overload

Role overload was measured using the 3-item role overload scale (Beehr et al., 1976).

#### Achievement Striving

Achievement striving was measured using the 10-item achievement striving sub-dimension of the NEO Personality Inventory (Costa and McCrae, 1985).

#### Agreeableness

Agreeableness was measured using the 4-item agreeableness subscale of the Mini-IPIP Scale (Donnellan et al., 2006).

#### Neuroticism

Neuroticism was measured using the 4-item neuroticism subscale of the Mini-IPIP (Donnellan et al., 2006).

#### Role Conflict

We measured work-personal conflict (WPC), personal-work conflict (PWC), family-personal conflict (FPC), and personal-family conflict (PFC) using the 5-item work-to-personal conflict scales (Wilson and Baumann, 2015). We measured work-family conflict (WFC) and family-work conflict (FWC) using the five-item WFC scales (Netemeyer et al., 1996).

### Results

#### Psychometric Properties

Confirmatory factor analyses (CFA) demonstrated that the items fit a unidimensional construct [χ^2^ (5) = 7.47, *p* < 0.01, CFI = 0.99, TLI = 0.99, RMSEA = 0.05, SRMR = 0.01]. Our five-item measure of the dispositional tendency to SOTT had a high reliability (α = 0.95). All of the five items had a factor loading of at least 0.86. Our measure showed moderate test-retest reliability (*r* = 0.61). Previous research on dispositional traits has shown that this level of correlation is high enough for stable traits measured over a similar period. For example, openness to experience has been shown to have a similar test-retest reliability (*r* = 0.67) over a one-month period (Donnellan et al., 2006).

#### Validity

We assessed the discriminant validity of our measure by examining latent correlations in a CFA model with items loading on their respective factors and correlations specified among those latent factors [χ^2^ (164) = 399.81, *p* < 0.01, CFI = 0.91, TLI = 0.89, RMSEA = 0.08, SRMR = 0.06]. This approach corrects for measurement error (Anderson and Gerbing, 1988; Hinkin and Schriesheim, 2008). As predicted, the dispositional tendency to SOTT was distinct from work role overload (*r* = 0.40), achievement striving (*r* = -0.05), and agreeableness (*r* = -0.04). In order to determine the criterion-related validity of our measure, we examined the correlations between our measure and each type of role conflict. Providing criterion-related validity and support for our substantive prediction that the tendency to SOTT predicts role conflict, the dispositional tendency to SOTT was significantly correlated with WPC (*r* = 0.42), PWC (*r* = 0.22), WFC (*r* = 0.40), family work conflict (*r* = 0.25), family personal conflict (*r* = 0.33), PFC (*r* = 0.18) – all of which were significant correlations.

To determine incremental validity, we assessed whether our measure predicted role conflict above and beyond known dispositional predictors. We entered known predictors of role conflict (i.e., neuroticism and negative affect) in Step 1 and the dispositional tendency to SOTT in Step 2. We also considered gender, number of children living at home, and tenure at current job as control variables, because previous research has shown they are related to our chosen outcomes (Frone et al., 1992; Byron, 2005). However, the results of the analyses did not change based on whether or not control variables were used, so we did not include control variables in the reported analyses. Results are shown in Table 2. The Δ*R*^2^ was significant for WPC (0.06), WFC (0.06), family work conflict (0.02), and family personal conflict (0.05). However, the Δ*R*^2^ was not significant for PFC or PWC. Thus, our scale displayed incremental validity for most, but not all, types of role conflict.

## Discussion

This project’s primary contribution is the development a self-report scale for a previously unmeasured dispositional tendency, the dispositional tendency to SOTT. This initial evidence suggests that our scale is valid, reliable, is distinct from other potentially similar constructs, and predicts several types of work-nonwork role conflicts above and beyond known dispositional predictors. Further validation is needed, but our scale shows promise as a measure of an important antecedent of work-nonwork role conflicts. Future research should consider whether this tendency is malleable. To the extent that it results from self-enhancement biases, it may be modifiable using decision-making strategies that are useful for mitigating the negative effects of self-enhancing biases in other domains (Dalal and Bolunmez, 2016). Given that our study only explored the impact of the dispositional tendency to SOTT on work-nonwork outcomes, future research should potentially explore how the dispositional tendency to SOTT may impact other outcomes (e.g., job performance, burnout). In the workplace, SOTT may even manifest in workaholism (Spence and Robbins, 1992). Furthermore, contextual variables such as work load, situational strength, and role clarity may be related to SOTT, and should be researched as such.

## Ethics Statement

This study was carried out in accordance with the recommendations of Institutional Review Board of George Mason university with written informed consent from all subjects. All subjects gave written informed consent in accordance with the Declaration of Helsinki. The protocol was approved by the George Mason University Institutional Review Board.

## Author Contributions

Both authors were involved in idea development and design of methodology. LB conducted data collection, data analysis, and wrote the manuscript. LK advised and edited the manuscript.

## Conflict of Interest Statement

The authors declare that the research was conducted in the absence of any commercial or financial relationships that could be construed as a potential conflict of interest.
